# Establishment of a leptospirosis model in guinea pigs using an epicutaneous inoculations route

**DOI:** 10.1186/1471-2334-12-20

**Published:** 2012-01-25

**Authors:** Yan Zhang, Xiao-Li Lou, Hong-Liang Yang, Xiao-Kui Guo, Xiang-Yan Zhang, Ping He, Xu-Cheng Jiang

**Affiliations:** 1 Department of Microbiology and Parasitology, Shanghai Jiao Tong University School of Medicine, Shanghai 200025, China; 2 Department of Center Laboratory, Songjiang Hospital Affiliated to First People's Hospital, Shanghai Jiao Tong University, Shanghai 201600, China; 3 Department of Microbiology, Immunology and Pathology, Colorado State University, Fort Collins, CO 80523-1682, USA; 4 Department of Pathology, Shanghai Jiao Tong University School of Medicine, Shanghai 200025, China

## Abstract

**Background:**

Leptospires are presumed to enter their host via small abrasions or breaches of the skin. The intraperitoneal route, although commonly used in guinea pig and hamster models of leptospirosis, does not reflect conditions encountered during natural infection. The aim of this study is to develop a novel leptospirosis guinea pig model through epicutaneous route and to elucidate the pathogenesis of leptospirosis in experimental guinea pigs by comparing the data from other studies using different infection routes.

**Methods:**

The guinea pigs were inoculated with 5 × 10^8 ^*Leptospira interrogans *strain Lai onto either shaved-only or abraded skin. The guinea pigs were sacrificed at 2, 8, 24, 48, 72, 96 and 144 h post-infection (p.i.) followed by harvest of the lungs, liver, kidneys, spleen, and the skin around the inoculated sites for further examinations. Hematoxylin and eosin (HE) staining and electron microscopy were used to detect the pathologic changes. Real time PCR and immunohistochemistry staining were performed to detect dynamic distribution of leptospires in blood and tissues, respectively.

**Results:**

In the guinea pigs with abraded skin inoculations, leptospires were detected in blood as early as 2 h post infection (p.i.) and then disseminated to the liver, lungs and kidneys of almost all animals within 96 h p.i.. Leptospires were also detected engulfed in the swelling vascular endothelial cells and were frequently aggregated around the capillaries in the dermis and subcutaneous tissue under the inoculated site. For the guinea pigs with abraded skin inoculations, hemorrhage at the dermis around the inoculated site was found before the appearance of internal organs hemorrhage, severe lesions such as hemorrhages in the lungs, nephritis, jaundice, haematuria were also observed, and two of seven guinea pigs died at 144 h p.i. while no lesions and leptospires were detected in the shaved-only guinea pigs using the same dose of strain Lai.

**Conclusion:**

Intact keratinocyte layer is a very efficient barrier against leptospires, and intact skin can prevent the infiltration of leptosipres to the host. Leptospires can penetrate abraded skin and quickly establish a systemic infection by crossing tissue barriers. We have successfully established a novel leptospirosis guinea pig model through epicutaneous inoculations route, which replicates a natural course of infection and appears to be an alternative way to investigate the pathogenesis of leptospirosis, especially in terms of early stage of host-pathogen interactions. This novel model may also be advantageous for studies of the mechanisms involved in cutaneous barriers and epidermal interactions with this organism.

## Background

Leptospirosis is a worldwide bacterial zoonosis caused by several species of invasive spirochetes belonging to the genus *Leptospira*. It affects humans in both rural and urban areas, particularly in developing countries with warm and humid climate [1-3]. Water contaminated by urine from animal reservoirs is the main source of human infection, usually through cut or abraded skin. Leptospirosis is characterized by a broad spectrum of clinical manifestations, ranging from subclinical infection to Weil's syndrome, a severe and potentially fatal disease characterized by hemorrhage, acute renal failure and jaundice [4]. Deaths may occur in less than 72 h after the advent of respiratory signs and symptoms such as severe hemorrhage of lungs, which usually appear between the fourth and the sixth day of disease [5].

The use of experimental models remains a critical component for elucidating pathogenesis of leptospirosis. Young guinea pigs and hamsters are the most commonly used experimental models for acute leptospirosis [6]. The intraperitoneal (i.p.) inoculation route is the most widely applied infection route by producing a lethal infection in experimental animals and mimicking the clinical symptoms of severe leptospirosis in humans [7-10]. However, this route of infection does not reflect real conditions encountered during natural infection, because leptospires are believed to enter the host via mucous membranes or abrasions of the skin. It has been a long time for researchers to challenge animals through alternative routes to mimic natural entry of leptospires into hosts. Even about one century ago, Ido and his colleagues attempted to reproduce natural conditions by conveying the leptospries directly to the guinea pig by the bite of rat (carrier of leptospires). The results indicated that leptospirosis is rarely transmitted by the bite of rat [11]. Since then, different infection routes such as conjunctival (c.j.) and subcutaneous (s.c.) have been employed in canine, horse, hamster and guinea pig, and resulting in acute leptospirosis in inoculated animals [11-16]. By using infection routes different from the classic i.p. inoculation, these studies contributed to the elucidation of pathogenesis of leptospirosis in experimental animals. However all these methods bypassed the epidermis of host, the entry route and mode of leptospires directly via epidermis have been poorly studied as of today. There is still little data on how the leptospires interact with the epidermis and if the inoculated leptospires can penetrate skin and disseminate in host subsequently.

In this study, we examined the ability of leptospires to produce infections in guinea pigs when applied to damaged or undamaged skin. The results showed that infection with virulent leptospires, using abraded skin inoculation route of infection, produced typical leptospirosis in guinea pigs, whereas there were no symptoms in guinea pigs through shaved-only skin. The availability of this novel model will enable understanding of the pathogenesis of leptospirosis, as well as to study cutaneous barriers and epidermal interactions with this organism.

## Methods

### Leptospiral strain and growth conditions

The *L. interrogans *serogroup Icterohaemorrhagiae serovar Lai strain Lai was obtained from the Institute for Infectious Disease Control and Prevention, Beijing, China. Virulence of leptospires was maintained by iterative passage in guinea pigs. Leptospires were grown in liquid Ellinghausen-McCullough-Johnson-Harris (EMJH) medium [17-19] at 28°C under aerobic conditions to the mid-log-phase and were counted using Petroff-Hausser counting chamber.

### Animals

Young male health guinea pigs, weighting 150-200 g each, were purchased from Institute of Biological Products of Shanghai. All guinea pigs were housed in specific cages containing autoclaved bedding, sterilized feed and water. The animal experiments were approved by the Animal Research Committee of Shanghai Jiao Tong University School of Medicine.

### Epicutaneous inoculation

Epicutaneous inoculation of *L. interrogans *on the flank skin of guinea pigs was performed using the modified procedure as previously described [20]. Briefly, the guinea pigs were carefully shaved over their left flank one day before inoculation, and then disinfected with iodine, washed with 75% alcohol followed by saline. The abraded skin was then prepared by gentle scraping with a surgical scalpel blade until a non-bloody glistening skin layer resulted, representing damage to the stratum corneum water barrier. An inoculum of 5 × 10^8 ^leptospires in 20 μl of sterile saline was added to 4 × 4 mm filter discs (Thermo Fisher Scientific, USA). The filter discs were then added on the either shaved-only or abraded skin. Sterile saline of 20 μl was applied on the abraded skin as negative control. Inoculated sites were covered with a 1.0 cm^2 ^piece of plastic sheet and overwrapped with Band-Aid waterproof tape (Johnson, USA). At different time points after inoculation, the number of leptospires left on the filter discs were checked by extensively PBS washing and then counted using Petroff-Hausser counting chamber. At 2 h post-infection (p.i.), there were about 5 × 10^4 ^leptospires left on the filter discs, suggesting that only one ten-thousandth of inoculums (5 × 10^4^/5 × 10^8 ^leptospires) have not penetrated to the skin. There was no leptospires detected after 24 h p.i.. It was supposed that all the leptospires were inoculated on the skin/or abrade skin at 24 h p.i.. Because guinea pigs were sacrificed at previously determined time points, the filter discs were removed after 2-24 h. Generally seven of leptospires infected abraded-skin guinea pigs, three of leptospires infected shaved-only guinea pigs and three of negative controls per time point were used in 3 independent experiments.

### Monitoring of infections

The guinea pigs were euthanized at 2, 8, 24, 48, 72, 96 and 144 h p.i.. Blood samples were collected by cardiac puncture for quantitative real-time PCR. The lungs, liver, kidneys, spleen, and the skin around the inoculated sites were harvested for histologic examinations. Paraffin sections were prepared and then stained with hematoxylin and eosin (HE). The rabbit antiserum specific to *L. interrogans *strain Lai was prepared in our lab using a modified procedure as previously described [21]. Immunohistochemistry staining was performed using the EnVision™ system (EnVision system, Dako, USA) [22]. In brief, paraffin-embedded tissue sections were dewaxed and rehydrated, treated with 3% H_2_O_2 _in methanol for 10 min, and then incubated in 0.1% trypsin at 37°C for 30 min. Sections were incubated in primary rabbit antibody (1:6000 dilution) specific for *L. interrogans *strain Lai for 12 h at 4°C, followed by EnVision™ for 30 min, then visualized with 3,3'-diaminobenzidine (DAB), and counter-stained with modified hematoxylin. Tissues for ultrastructural studies using a modified procedure as previously described [9]. Briefly, tissues around the wound were removed and fixed in 2% glutaraldehyde immediately for 24 h, then post-fixed in 1% osmium tetroxide, dehydrated in graded ethanols, and embedded in Epon 618. Ultrathin (70 nm) sections were stained with uranyl acetate and lead citrate, and examined with a PHILIP CM-120 electron microscope.

### Blood DNA extraction and the following real-time PCR

Genomic DNA of leptosipres from blood samples was extracted using a blood DNA purification kit according to manufacturer's instruction (Omega, USA). The concentration of leptospires in animal blood was quantified by real-time PCR using Applied Biosystems 7500Fast. All reactions were performed with the Power SYBR Green PCR Master Mix (Applied Biosystems, USA). The 116 bp 16S rRNA gene amplicons of leptospires were quantified using the primers F (5'-TCC TGG CTC AGA ACT AAC GC-3'), and R (5'-TCC CAG ACT CAG AGG AAG AT-3'). PCR conditions were as follows: initial denaturation at 95°C for 10 min, followed by 40 cycles of amplification 95°C for 15 s and 60°C for 60 s. The standard curve for quantification was made using a modified procedure as previously described [23]. In brief, the 116 bp 16S rRNA gene amplicons were cloned into the vector pMD19 by using the TA cloning kit (Invitrogen, USA). There are two copies of the 16S rRNA gene per *Leptospira*. The 10-fold dilutions of recombinant plasmid with 16S rRNA was used to establish the standard curve for quantification. Results were expressed as the number of leptospires in 1 μl blood. The real-time PCR was performed in duplicate for each DNA extraction. Three guinea pigs were used for each time point in each group.

## Results

### General observations

**Figure 1:**
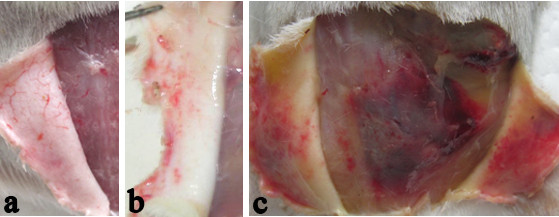
**Macroscopic examination of dermis and muscles around the site-inoculation of guinea pigs**. Guinea pigs with leptospires inoculation on abraded skin with clinical findings of hemorrhage on the dermis at 24 h p.i. (**b**) and hemorrhage and jaundice on the dermis and muscles at 144 h p.i. (**c**) that are absent in guinea pigs with leptospires inoculation on shaved-only skin (**a**).

**Figure 2:**
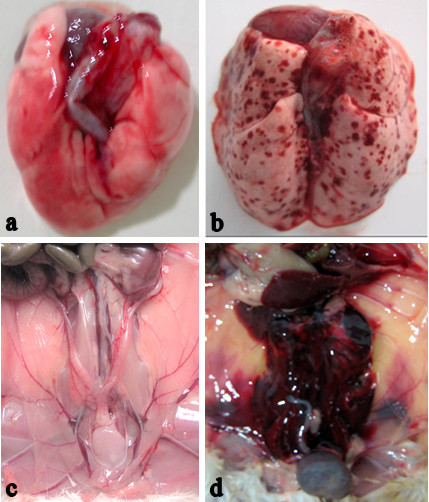
**Macroscopic examination of guinea pigs with epicutaneous inoculation of *L. interrogans***. Lungs (**a**), peritoneal surfaces and kidney (**c**) of guinea pigs with leptospires inoculation on shaved-only skin did not exhibit hemorrhage, in contrast with lungs (**b**), peritoneal surfaces and kidney (**d**) of guinea pigs with leptospires inoculation on abraded skin. Tissues were observed at 144 h p.i.

In order to determine if the inoculated leptospires can penetrate the skin and disseminate in host subsequently, guinea pigs were epicutaneously inoculated onto either shaved-only or abraded skin with 5 × 10^8 ^leptospires. Guinea pigs were then monitored daily for appearance of clinical symptoms. The results revealed that inoculation of *L. interrogans *onto shaved-only skin of guinea pigs did not show any signs of infection by 144 h p.i. (Figure 1a). In contrast, inoculation onto abraded skin of guinea pigs developed clinical signs, including evidence of ruffled hair coat, listlessness and isolation within 72 h p.i.. Jaundice was present in skin after 96 h p.i. (Figure 1c). All of the guinea pigs with skin abraded inoculations developed severe hemorrhages in lungs, peritoneal surfaces and kidney at 144 h p.i. (Figure 2b and 2d), and two out of seven guinea pigs died at 144 h p.i. (Additional file 1: Table S1). Because guinea pigs were killed at previously determined time points, it is not known whether the severely ill guinea pigs killed on 72, 96 and 144 h p.i. would have survived.

### Occurrence of hemorrhage after inoculation

**Figure 3:**
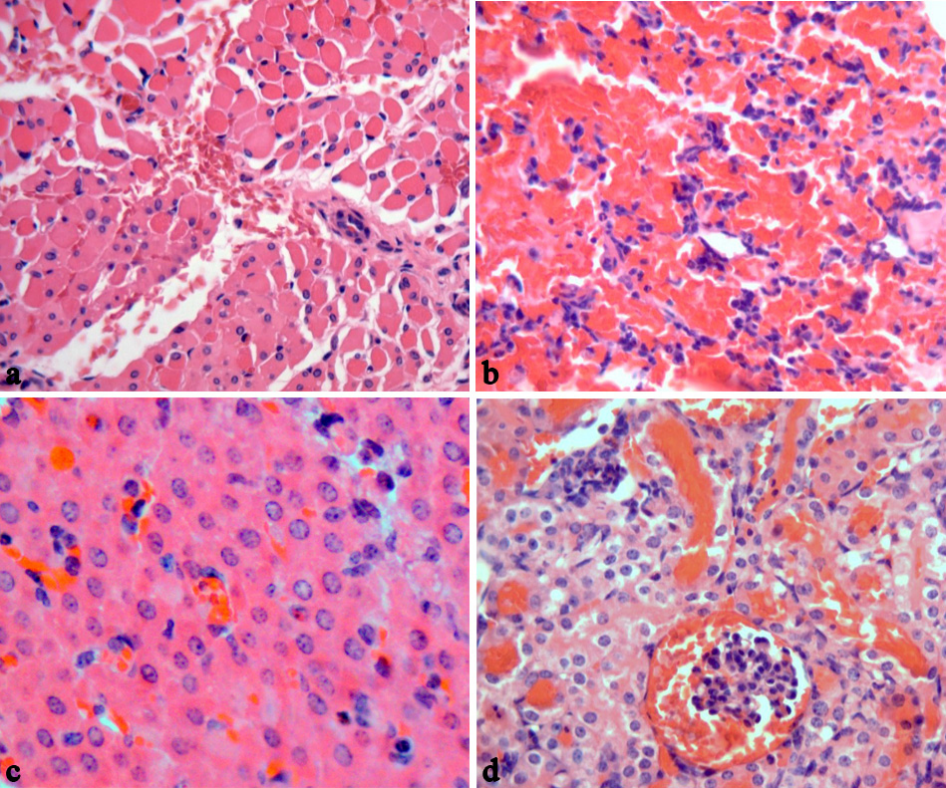
**Pathologic changes of guinea pigs with leptospires inoculation on abraded skin**. HE staining of muscular layer around the site-inoculation (**a**), lung (**b**), liver (**c**) and kidney (**d**) of guinea pigs with leptospires inoculation on abraded skin. Images are from guinea pigs at 24 h p.i. (**a**) and 144 h p.i. (**b**, **c**, **d**). (Magnification, × 400).

All of the guinea pigs with abraded skin leptospires inoculation showed spotted hemorrhage at the dermis around the site-inoculation as early as 24 h p.i. (Figure 1b). The hemorrhage, characterized by petechia and confluent patchy, spreaded from inoculated site to subcutaneous tissue and muscular layer and progressed with time. HE staining of muscular layer showed a few of erythrocytes distributed among muscle bundle at 24 h p.i. (Figure 3a) and became more severe, progressing to a large number of erythrocytes filled with muscle fibers at 144 h p.i.. Almost all of the guinea pigs with abraded skin inoculation developed hemorrhages in lungs after 72 h p.i. (6/7 at 72 h p.i., 5/7 at 96 h p.i., 7/7 at 144 h p.i., Additional file 1: Table S1). The alveolar hemorrhage appeared as small foci at 72 h p.i., and developed to coalesced areas of hemorrhage at 96 and 144 h p.i. as severity increased (Figure 3b). HE staining demonstrated focal hemorrhage, interstitial edema and necrotic foci in liver tissue after 96 h p.i. (Figure 3c). Extensive hemorrhage on the kidney and peritoneal surfaces could be seen at 96-144 h p.i. (Figure 2). The kidney changes occurred in tubular epithelial cells, characterized by acute tubular necrosis, with the renal tubules filled with erythrocytes (Figure 3d). Almost all of the guinea pigs had haematuria after 96 h p.i. (5/7 at 96 h p.i., 7/7 at 144 h p.i., Additional file 1: Table S1).

In contrast with progressing and severe leptospirosis in guinea pigs with the abraded skin leptospires inoculation, none of these features was observed in the shaved-only and saline inoculated control animals (Figure 1 and 2).

### Leptospires distribution in blood and tissues of infected guinea pigs

**Figure 4:**
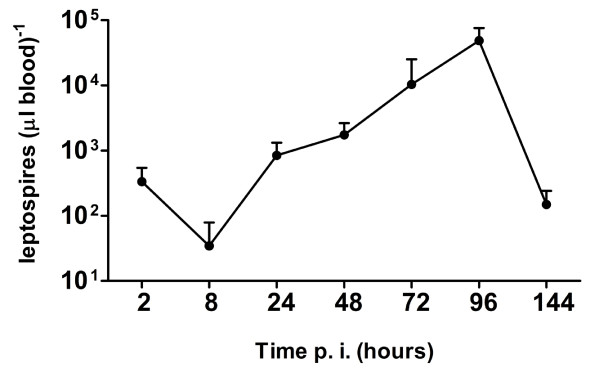
**Variation of *L. interrogans *burdens in the blood of infected guinea pigs with abraded skin inoculation**. Leptospires burdens in groups of infected guinea pigs with abraded skin inoculation were analyzed by real-time PCR at 2, 8, 24, 48, 72, 96 or 144 h p.i. by measuring copies of the 16S rRNA gene. Data represent means ± standard errors of the levels of leptospires in blood; results are from 3 animals per point, tested in 3 experiments.

**Figure 5:**
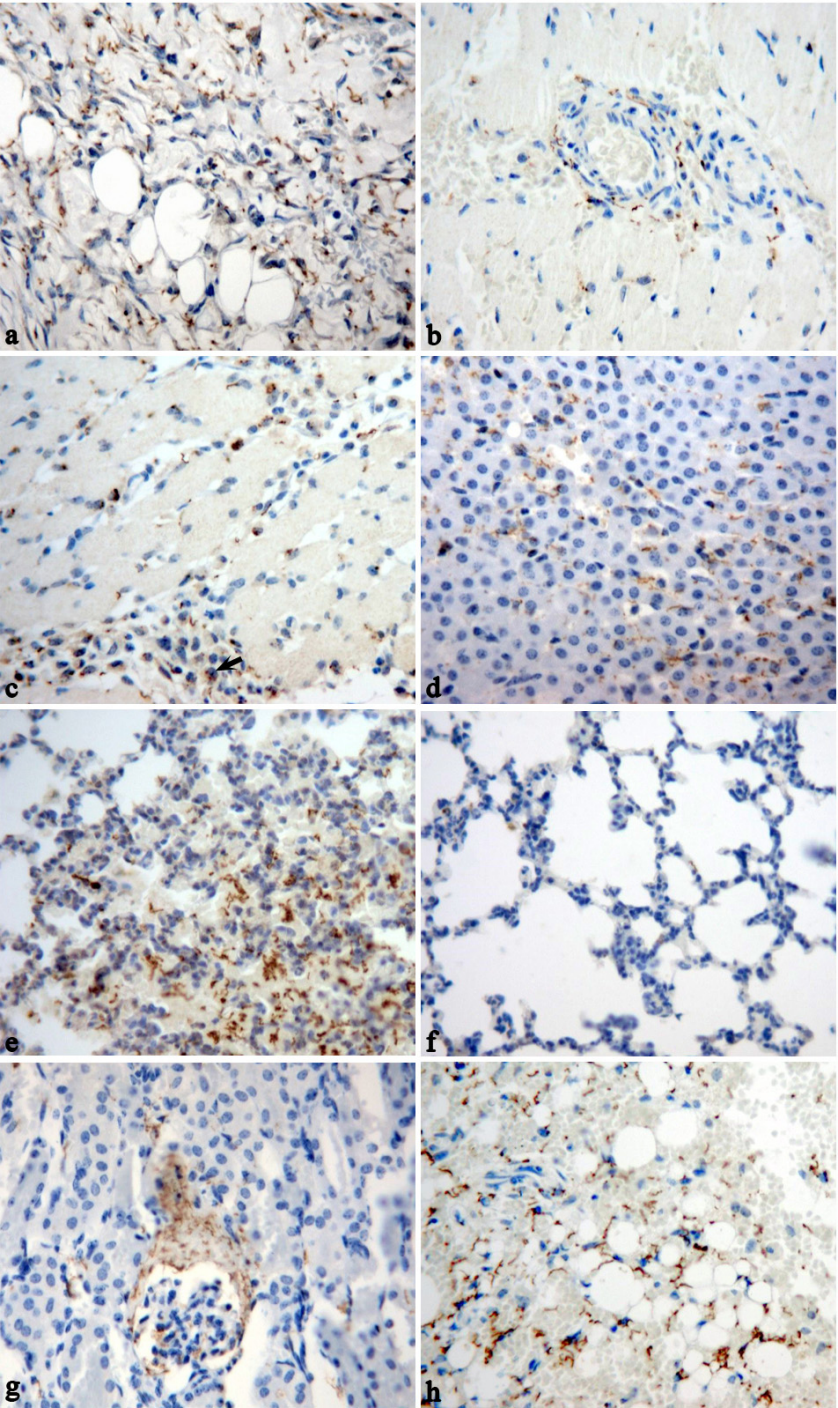
***L. interrogans *burdens in various tissues of guinea pigs with abraded skin inoculation**. Visualization of leptospires (brown particles and threads) in the dermis and subcutaneous (**a**), muscular layers (**b **and **c**), liver (**d**), hemorrhagic area of lungs (**e**), none hemorrhagic area of lungs (**f**), kidney (**g**) and retroperitoneum (**h**) of guinea pigs with leptospires inoculation on abraded skin. Black arrow indicates leptospires in the cytoplasm of phagocytes. Tissues were observed at 2 h p.i. (**a**), 24 h p.i. (b and c) and 144 h p.i. (**d-h**). Leptospires were stained by immunohistochemistry. (EnVision, magnification, × 400).

**Figure 6:**
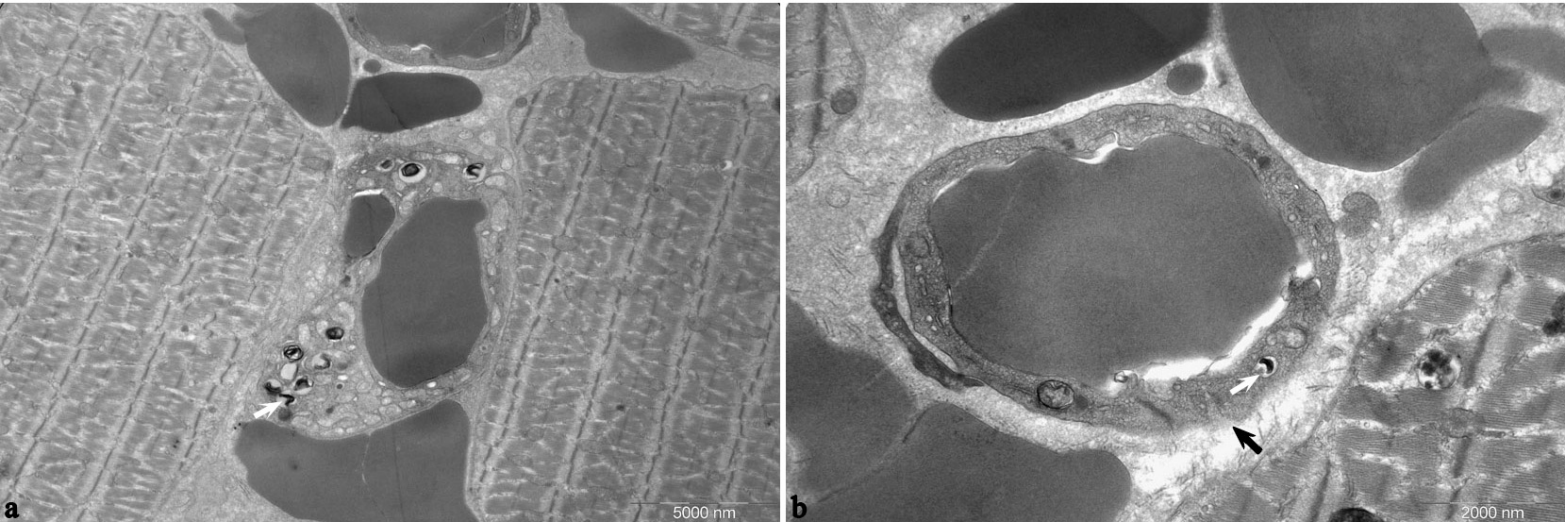
**Electron photomicrographs of muscular layers around the site-inoculation of guinea pigs with leptospires inoculation on abraded skin at 144 h p.i**. Leptospires were detected in the cytoplasm of vascular endothelial cells (indicated by white arrows, a and b). The damaged vascular basement membrane indicated by black arrow (**b**). Magnification is at 5800 (**a**) and 13500 (**b**).

Blood and tissue samples were processed for real time PCR and immunohistochemistry staining, respectively. In the guinea pigs with abraded skin inoculation, leptospires were detected in blood as early as at 2 h p.i. with approximately 3 × 10^5 ^leptospires ml^-1^. Levels of leptospires in blood dropped by 1 log at 8 h p.i., and then increased in the blood between 8 and 96 h p.i.. The bacteraemia peaked at 96 h p.i. with approximately 5 × 10^7 ^leptospires ml^-1 ^and then quickly decreased by more than 2 logs at 144 h p.i. (Figure 4). In the guinea pigs with abraded skin inoculation, leptospires were seen to have invaded from the inoculated site into the dermis and subcutaneous by immunohistochemistry as early as at 2 h p.i. (Figure 5a). At 48 h p.i., abundant leptospires were detected in the dermis and subcutaneous of hemorrhagic area and were rarely detected in adjacent none hemorrhagic areas (data not shown). In muscular layer, leptospire were detected aggregated around the capillaries at 24 h p.i. and were frequently phagocytized by neutrophils and macrophages (Figure 5b and 5c). Leptospires were also detected engulfed in the swelling vascular endothelial cells by transmission electron microscope, and the damaged vascular basement membrane was observed (Figure 6). In addition, a pronounced acute inflammatory response were induced in site inoculated and surrounding tissues characterized by the presence of neutrophils, macrophages, lymphocytes and histiocytes at 48 h p.i.. At the late stage (from 72 to 144 h p.i.), inflammatory cells in the subcutaneous tissue and muscular layers decreased gradually, and replaced by fibroblasts proliferation. In liver, thread-like leptospires were predominantly found closely associated with hepatocyte as well as granular deposits in Kupffer cells after 48 h p.i. (Figure 5d). Lungs of infected guinea pigs showed significant microscopic hemorrhage with intact leptospires in every hemorrhagic area detected by immunohistochemistry, whereas rare leptospires could be detected in none hemorrhagic areas (Figure 5e and 5f). In the kidney, large numbers of typical leptospires were seen in the cavity of glomerular capsules after 96 h p.i. (Figure 5g). Linear leptospires were discovered surrounding the peritoneal capillaries after 72 h p.i. (Figure 5h). Lesions and leptospires were not detected in the shaved-only and saline inoculated control guinea pigs (data not shown).

## Discussion

Intraperitoneal injection is the most widely used infection route in experimental leptospirosis studies [9,24]. It reproduces the processes of the human leptospirosis in the animal models using a very easy way. However, intraperitoneal injection does not reflect the natural transmission of the pathogen. Leptospires are thought to enter the human body via cuts or abrasions in the skin. The entry of leptospires directly via epidermis has been poorly studied. Some reports of clinical leptospirosis cases have clearly identified the initial cutaneous injury [25], others have not noted such a preexistent lesion [26,27]. It is not known whether the organism can penetrate intact skin or abraded skin. In this study, we established a guinea pigs leptospirosis model using epicutaneous inoculations route, to gain a better understanding of host-pathogen interaction and the pathogenesis of leptospirosis.

In this study, guinea pigs were inoculated with leptospires onto either shaved-only or abraded skin. Guinea pigs with abraded skin displayed clinical signs of leptospirosis. In contrast, lesions were not detected in the shaved-only animals which were inoculated the same amount of virulent *L. interrogans *strain Lai. These data confirmed that the intact keratinocyte layer is a very efficient barrier against leptospires, and intact skin can prevent the infiltration of leptosipres to the host.

It should be noted that the *L. interrogans *strain Lai used in this research was originated from a female patient who died of pulmonary hemorrhage after an infection with this organism, which had previously been studied in a variety of animal models and found to develop a typical leptospirosis in guinea pigs with intraperitoneal route [9,28,29]. The inoculation dose was referred to the previous guinea pig model reported in our lab [9].

Our study here showed that infection with the *L. interrogans *strain Lai using abraded skin inoculation route of infection produced a lethal infection in guinea pigs that mimicked the clinical characteristics of severe leptospirosis in patients, as described elsewhere [5,30,31]. The main clinical signs were serious pulmonary hemorrhage, jaundice, retroperitoneal hemorrhage and renal hemorrhage.

Our data showed that virulent leptospires can rapidly (within 2 h) penetrate the abraded epidermis and enter the dermis; at some point within 2 h p.i., the invading organisms also distribute to blood. Attachment to host cells and host extracellular matrix (ECM) components is likely the necessary step for leptospires to penetrate, disseminate and persist in mammalian host tissues. Consistent with the ability of *L. interrogans *to migrate through host tissues, a wide range of adhesion molecules were discovered in these organisms that may facilitate this process [32-34]. It has been reported that many leptospiral proteins, including LigA/B, Lsa21, Lsa27, LenA to F, LipL32, OmpL37, TlyC and LipL53, have affinity for ECM and cell surface in vitro [32-41]. Some of these proteins, such as OmpL37, were reported to have the strong binding affinity for skin and aorta elastin, and might facilitate the attachment of leptospires to elastin-rich inner layer of the skin as well as vascular structures [37].

It is evident that leptospires penetrate abraded skin and quickly establish a systemic infection by crossing tissue barriers. It is likely that *L. interrogans *can move through the tissue barrier by association with blood vessels, because leptospires were detected aggregated around the capillaries in muscular layer and peritonaeum in this study. It was thought that leptospires, like other spirochaetes, spread through intercellular junctions [42]. However, they have been shown to efficiently enter host cells in vitro [43-45]. Previous work accomplished by Martinea-Lopez *et al*. demonstrated that *L. interrogans *can disrupt the dynamics of the actin cytoskeleton in the human microvascular endothelial cell line and rapidly translocate through the cell layers [46]. Other studies in human leptospirosis have shown that leptospiral antigens were detected in the cytoplasm of the endothelial cells of septal capillaries [30]. Consistent with these results, leptospires were detected intracellularity in the vascular endothelial cells and disruptions of vascular basement membrane were also observed in this study. Our findings suggested that leptospires cross endothelial barrier and cause heamatogenous dissemination by pass through the endothelial cell cytoplasm.

When *L. interrogans *strain Lai was inoculated on the abraded skin, localized changes around the inoculated site were detected. All of the guinea pigs showed hemorrhage at the dermis around the site-inoculation before the appearance of internal organs hemorrhage. Skin hemorrhage was rarely reported in animals infected experimentally through the i.p. route, and little attention has been called for. The mechanism of hemorrhage caused by leptospirosis has not been elucidated yet. Factors contributing to the hemorrhage might involve direct action of toxins and autoimmune process. Nicodemo and coworkers detected the intact leptospires in capillary endothelial cells, indicating the lung injury is directly triggered by leptospires and/or by their toxic products [30]. Another study demonstrated the deposition of antibodies and complement along the alveolar basement membrane of infected guinea pigs, indicating pulmonary hemorrhage might be led by autoimmune process [8]. Our data showed that abundant leptospires were detected in the dermis and subcutaneous tissue of hemorrhagic area and were rarely detected in adjacent none hemorrhagic areas, confirming the high burden of leptospires in the dermis is an important factor to cause hemorrhage. Humoral immune response seems not be associated with the pathogenesis of skin hemorrhage, as dermis hemorrhage developed as early as 8-24 h p.i.. Further examination of the local hemorrhage may give a clue to understand the mechanism of hemorrhage in this disease.

Hemorrhage in the skin is produced as one of the general symptoms in clinical cases [4]. However, Hemorrhage localized at infected site was rarely recognized clinically. Local hemorrhage in our experiment model might caused by high dose inoculation of leptospires. When in nature infection, it seems like that low dose leptospires in the cuts or abrade skin will not cause skin hemorrhage until large amount of pathogen proliferated in the circulation, and then extensive skin hemorrhage will be produced.

Recently, Lourdault and his colleagues compared different routes (i.p., c.j. and s.c. inoculation) of infection and the dissemination of leptospries in blood and tissues of guinea pigs using multiple methods including real-time PCR [16]. The results showed infected guinea pigs developed similar physical signs and pathological changes after i.p., s.c. and c.j. inoculation with leptospires, and the bacterial burden in tissues and histopathology revealed no major differences between the three routes of infections [16]. In the guinea pigs with abraded skin inoculation, our real-time PCR results showed that the bacteraemia peaked at 96 h p.i. and then quickly decreased at 144 h p.i., which were consistent with the result of i.p. inoculated guinea pigs or s.c. inoculated hamsters reported by Lourdault and Truccolo respectively [15,16]. It is interesting to note that the high leptospires burden (3 × 10^5 ^leptospires ml^-1^) detected in the blood at 2 h p.i., and then quickly dropped by 1 log at 8 h p.i.. It is speculated that high dose (5 × 10^8^) leptospires inoculation cause a rapid flood of leptospira from the inoculated site to the bloodstream, then the majority of the leptospires were cleared by the innate immune system in the following several hours. As pathogenic *Leptospira *were reported to be able to survive, and be more resistant to the action of the complement system [47-49], polymorphonuclear neutrophils (PMNs), which constitute the largest population of intravascular phagocytes, are expected to play an important role in leptospiral clearance. It was reported that PMNs are able to kill pathogenic strains of *Leptospira *by oxygen dependent and independent mechanisms [50]. However, some experimental models showed that phagocytosis of pathogenic *Leptospira *by neutrophils and macrophages is only effective if this pathogen is opsonized by specific IgG [51-53]. Further investigations on PMNs activation and elimination of pathogenic leptospires are required to elucidate the establishment of innate immune responses in leptospirosis.

The traditional intraperitoneal inoculation is easy to handle and allows reproducible amounts of leptospires to be introduced. It is still the most widely used model to study the systemically infection of leptospirosis. However, there are some shortages of i.p. or other non-epicutaneous routes when apply on the pathogens causing infection through skin. In study performed by Bischof and colleagues, the subcutaneous injection of *B. anthracis *(Sterne strain, which lacks the pX02 capsule plasmid) caused lethal infection in C57BL/6 mice, while quite resistant to epicutaneous inoculation of *B. anthracis *onto abraded skin [20]. This study suggested that our epicutaneous inoculation model would be an alternative way to apply the characterizations of *Leptospira *mutants that are deficient in protein with binding affinity for skin.

## Conclusion

In summary, our current research demonstrated *L. interrogans *strain Lai had the ability to penetrate lesional epidermis after epicutaneous inoculation in guinea pigs, and also had the ability to disseminate systemically from the skin within 48 h of such inoculation. The guinea pigs leptospirosis model with an epicutaneous inoculation route described here replicated a natural course of infection and revealed epicutaneous inoculation might be an alternative route to investigate the pathogenesis of leptospirosis, especially when focus on the early steps of infection while the intraperitoneal inoculation is still a classic and main infection route due to its easy to handle feature. Our current model may also contribute to gain a better understanding of the mechanisms involved in cutaneous barriers and epidermal interactions with this organism, and consequently a delineation of the host-bacterium relationship with the aim of establishing prevention, early diagnosis, and efficient therapeutic regimens.

## Competing interests

The authors declare that they have no competing interests.

## Authors' contributions

YZ, PH and XCJ designed the research project. YZ and XYZ coordinated the leptospira culture. YZ and XLL participated in developing a guinea pigs model of leptospirosis and pathology experiments. PH and XLL carried out the real-time PCR experiments. XCJ and HLY examined tissue samples. PH, HLY and XKG drafted the manuscript. All authors contributed to the writing and preparation of the manuscript. All authors read and approved the final manuscript.

## Pre-publication history

The pre-publication history for this paper can be accessed here:

http://www.biomedcentral.com/1471-2334/12/20/prepub

## Supplementary Material

Additional file 1 **Table S1**. leptospires burdens and lesions in guinea pigs with leptospires inoculation on abraded skin. Click here for file
