# Supplementary material for: Establishment of a leptospirosis model in guinea pigs using an epicutaneous inoculations route
Source: BMC Infect Dis. 2012 Jan 25;12:20. doi: 10.1186/1471-2334-12-20 (PMC3329641; doi:10.1186/1471-2334-12-20)
Supplement: Additional file 1 — Table S1. leptospires burdens and lesions in guinea pigs with leptospires inoculation on abraded skin. [file 1471-2334-12-20-S1.DOC]

Supplemental table 1: leptospires burdens and lesions in guinea pigs with leptospires inoculation on abraded skin*

| Hours | Detection of leptospires in tissues  (NO. positive / NO. tested) | | | | | | Hemorrhage in tissues  (NO. positive / NO. tested) | | | | | | | Jaundice  (NO. positive /  NO. tested) | Mortality  (NO. died /  NO. tested) |
| --- | --- | --- | --- | --- | --- | --- | --- | --- | --- | --- | --- | --- | --- | --- | --- |
|  | Dermis | Muscular layer | Liver | Lung | Kidney | Retroperitoneum |  | Dermis | Muscular layer | Liver | Lung | Kidney | Retroperitoneum |  |  |
| 2 | 7/7 | 0/7 | 0/7 | 0/7 | 0/7 | 0/7 | 0/7 | | 0/7 | 0/7 | 0/7 | 0/7 | 0/7 | 0/7 | 0/7 |
| 8 | 7/7 | 0/7 | 0/7 | 0/7 | 0/7 | 0/7 | 2/7 | | 0/7 | 0/7 | 0/7 | 0/7 | 0/7 | 0/7 | 0/7 |
| 24 | 7/7 | 7/7 | 0/7 | 0/7 | 0/7 | 0/7 | 7/7 | | 5/7 | 0/7 | 0/7 | 0/7 | 0/7 | 0/7 | 0/7 |
| 48 | 7/7 | 7/7 | 4/7 | 0/7 | 0/7 | 0/7 | 7/7 | | 6/7 | 0/7 | 0/7 | 0/7 | 0/7 | 0/7 | 0/7 |
| 72 | 7/7 | 7/7 | 6/7 | 6/7 | 0/7 | 3/7 | 7/7 | | 7/7 | 0/7 | 6/7 | 0/7 | 3/7 | 0/7 | 0/7 |
| 96 | 7/7 | 7/7 | 7/7 | 5/7 | 5/7 | 5/7 | 7/7 | | 7/7 | 3/7 | 5/7 | 5/7 | 5/7 | 5/7 | 0/7 |
| 144 | 7/7 | 7/7 | 7/7 | 7/7 | 7/7 | 7/7 | 7/7 | | 7/7 | 6/7 | 7/7 | 7/7 | 7/7 | 7/7 | 2/7 |

* NO. positive = numerus positive; NO. tested = numerus tested;
